# Supplementary figures and images for: The association between circadian syndrome and possible sarcopenia in an aging population: A 4-year follow-up study
Source: PLoS One. 2025 May 13;20(5):e0323211. doi: 10.1371/journal.pone.0323211 (PMC12074653; doi:10.1371/journal.pone.0323211)

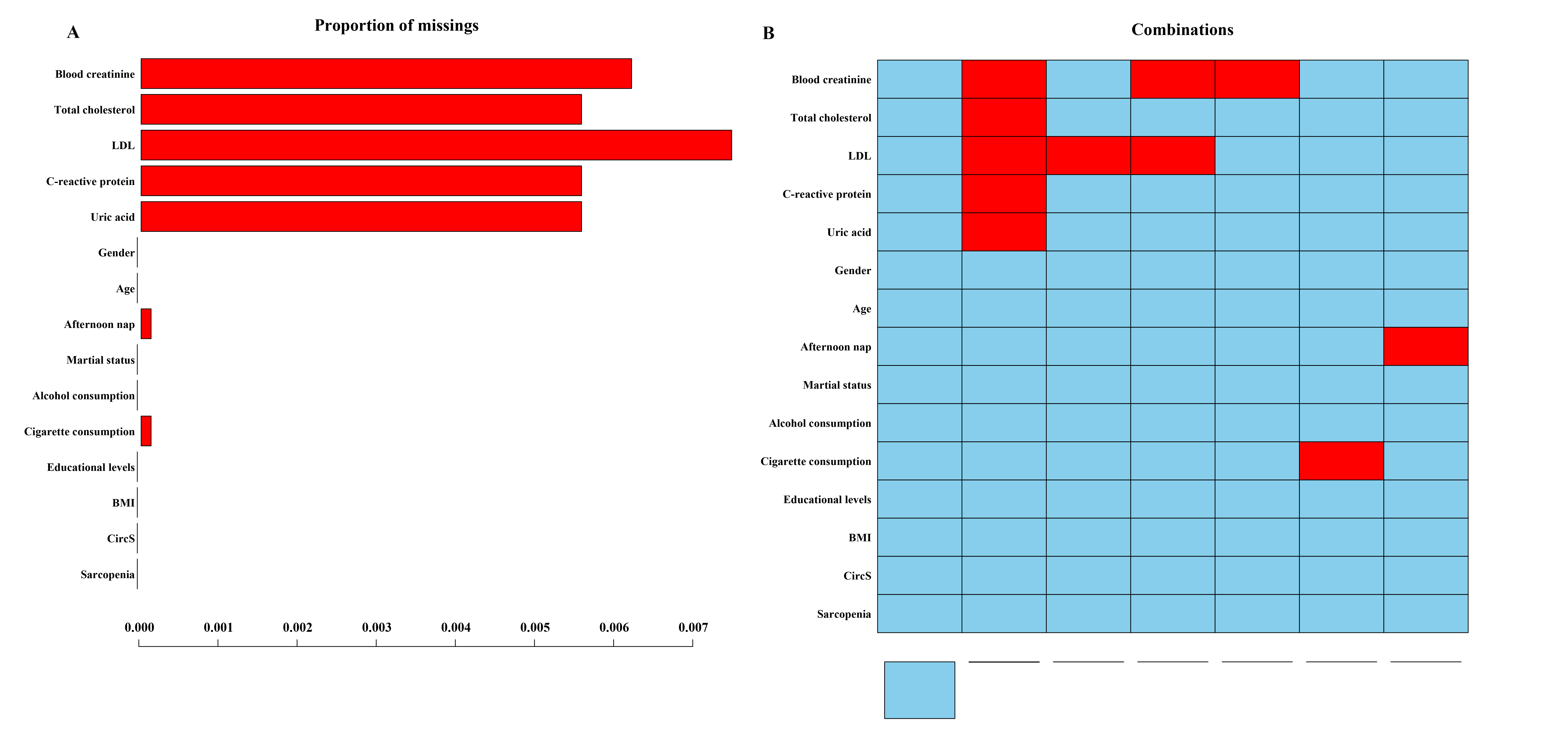

Supplement: S1 Fig — A shows the percentages of missing values of covariates. B shows the combinations of missing values of covariates. (JPG) [file pone.0323211.s001.jpg]

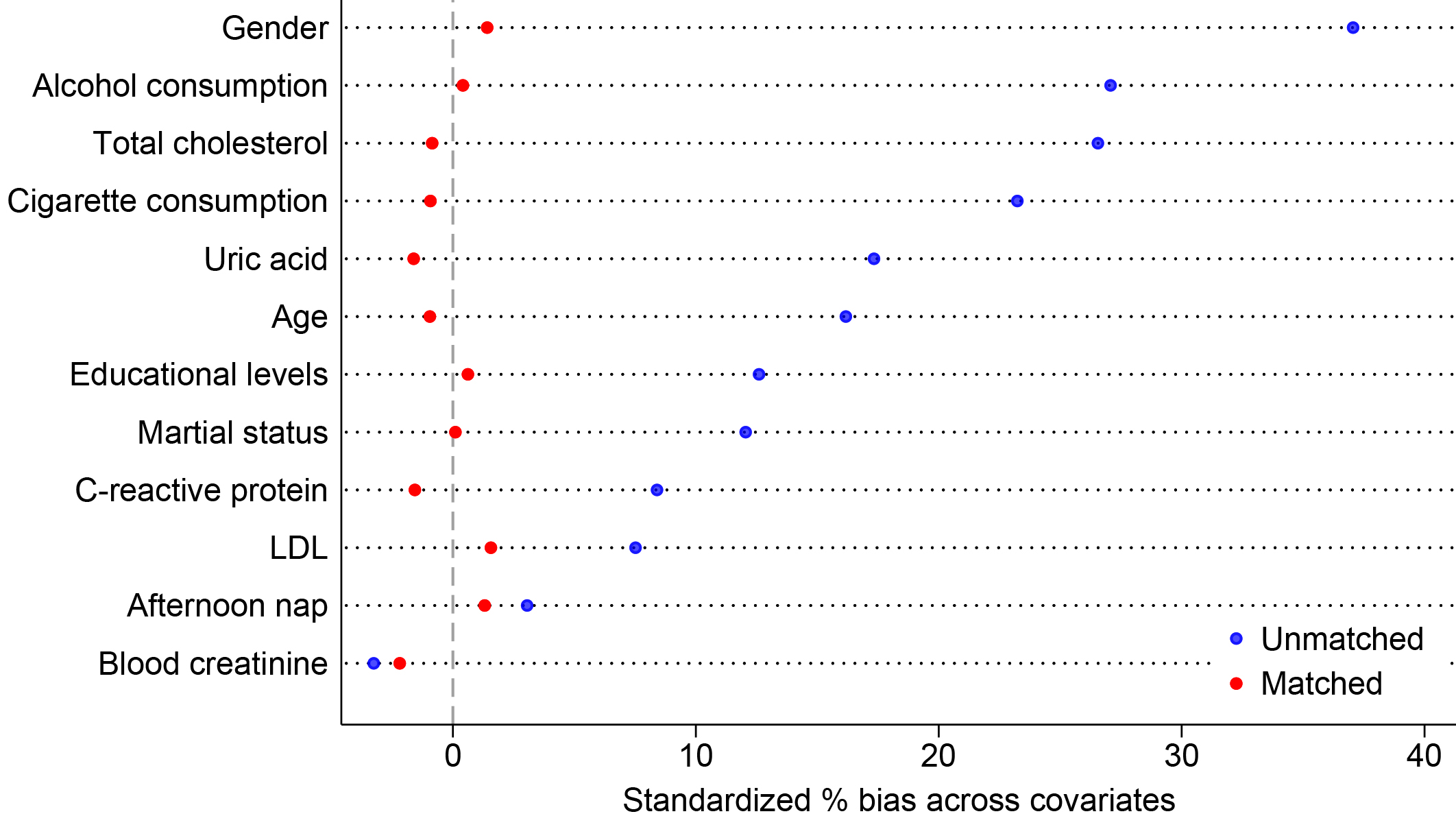

Supplement: S2 Fig — Absolute standardized bias < 10% is accepted as a well-balanced dataset between covariates. In this figure, all the biases were close to null after PSM. (JPG) [file pone.0323211.s002.jpg]

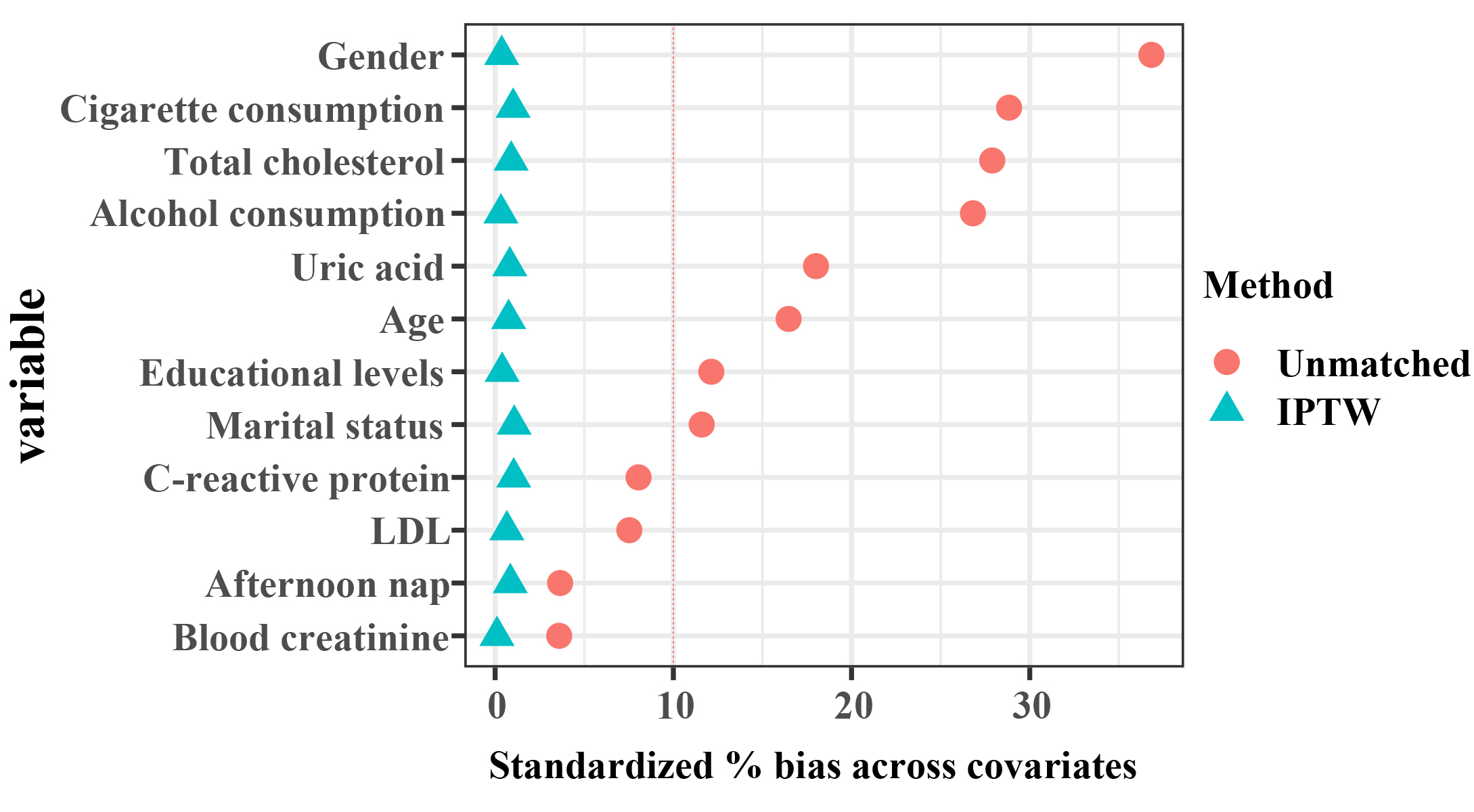

Supplement: S3 Fig — Absolute standardized bias < 10% is accepted as a well-balanced dataset between covariates. In this figure, all the biases were close to null after IPTW. (JPG) [file pone.0323211.s003.jpg]
